# Twenty-Seven Y-Chromosome Short Tandem Repeats Analysis of Italian Mummies of the 16th and 18th Centuries: An Interdisciplinary Research

**DOI:** 10.3389/fgene.2021.720640

**Published:** 2021-09-30

**Authors:** Carla Bini, Elisabetta Cilli, Stefania Sarno, Mirko Traversari, Francesco Fontani, Alessio Boattini, Susi Pelotti, Donata Luiselli

**Affiliations:** ^1^Laboratory of Forensic Genetics, Department of Medical and Surgical Sciences, University of Bologna, Bologna, Italy; ^2^Laboratory of Ancient DNA (aDNALab), Department of Cultural Heritage, University of Bologna, Ravenna, Italy; ^3^Laboratory of Molecular Anthropology and Centre for Genome Biology, Department of Biological, Geological and Environmental Sciences, University of Bologna, Bologna, Italy

**Keywords:** ancient DNA, Italian mummies, forensic genetics, anthropology, Y-chromosome STRs

## Abstract

Roccapelago (MO) is a small village located in the Northern Central Apennines, with a population of 31 inhabitants (2014). In 2010, more than 400 individuals dated between the end of the 16th and the 18th century, many of which partially mummified, were discovered in the crypt of the church. This small village, because of its geographical location and surrounding environment, seems to possess the characteristics of a genetic isolate, useful for population genetics and genealogical analyses. Thus, a diachronic study of DNA aimed at investigating the structure and dynamics of the population of Roccapelago over the about 4 centuries, was conducted by analyzing ancient and modern inhabitants of the village. The 14 modern samples were selected by considering both the founder surnames of the village, identified thanks to the study of parish registers, and the grandparent’s criterion. From 25 ancient mummies, morphologically assigned to male individuals, the petrous bone, that harbors high DNA amounts, was selected for the DNA extraction. The quantification and qualitative assessment of total human male DNA were evaluated by a real-time PCR assay using the Quantifiler Trio DNA Quantification Kit and multiplex PCR of 27 Y-chromosome short tandem repeat (Y-STR) markers included in the Yfiler Plus PCR Amplification Kit, with seven rapidly mutating Y-STR loci for improving discrimination of male lineages, was performed to genotype the samples. Y-STRs were analyzed according to the criteria of ancient DNA (aDNA) analysis to ensure that authentic DNA typing results were obtained from these ancient samples. The molecular analysis showed the usefulness of the Y chromosome to identify historically relevant remains and discover patterns of relatedness in communities moving from anthropology to genetic genealogy and forensics.

## Introduction

Remains of ancient people represent an invaluable opportunity to understand patterns of past population dynamics, appearance and distribution of phenotypes, familial relationships, sex determination, and spread of diseases. They also constitute the opportunity to apply and test protocols, combining and optimizing the archaeogenetic and the forensic ones, in order to achieve and improve results in both fields.

**Figure 1:**
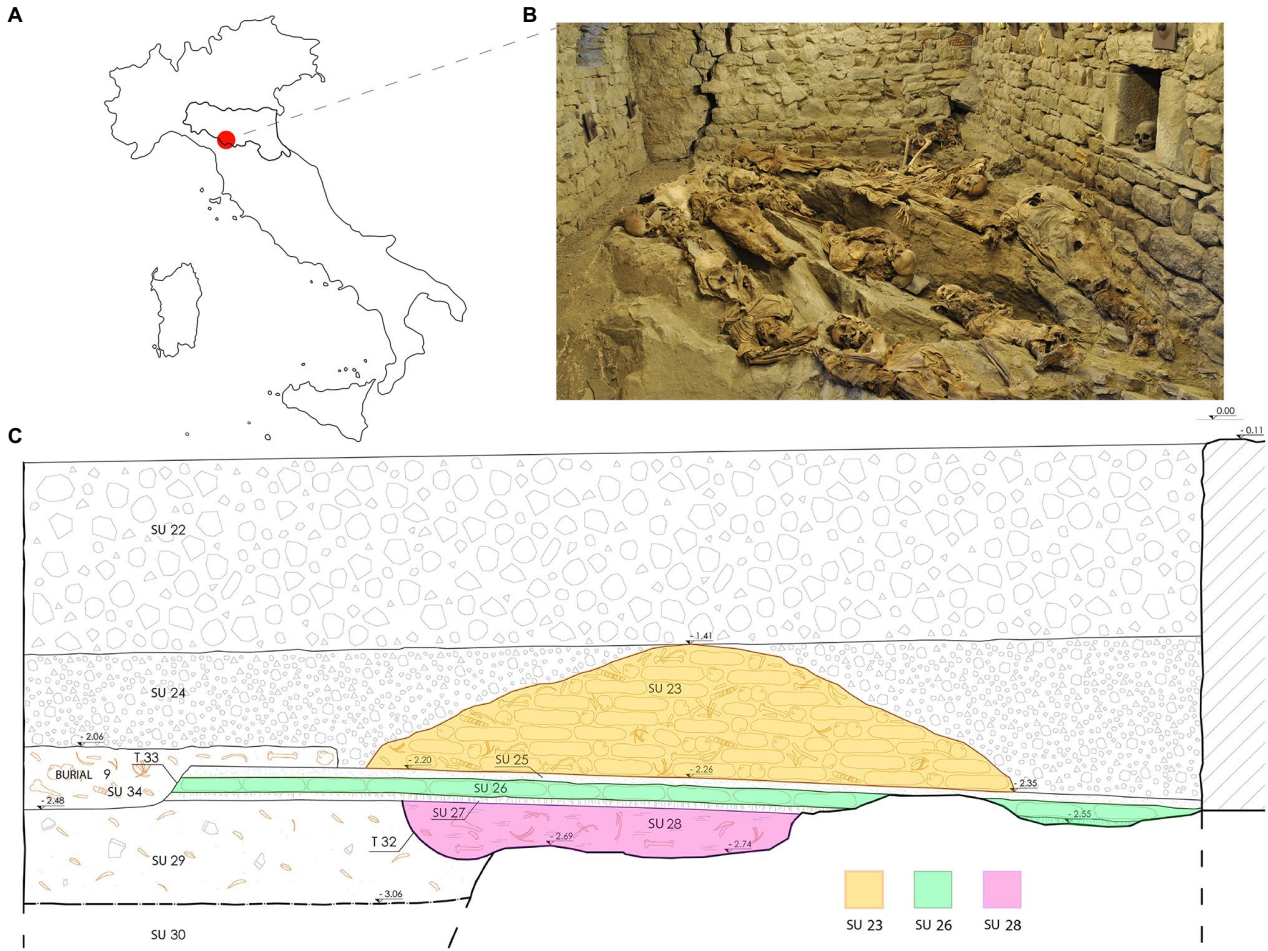
**(A)** The geographical location of Roccapelago; **(B)** an image of the current musealization inside the crypt; **(C)** graphical section of the stratigraphic units (SU) of the Roccapelago crypt. The SU interested by the sampling are labeled with different colors. Modified from the original illustration made by Alessandra Alvisi.

In the context of studies about past populations, the Italian Apennines kept a precious witness of its past. In fact, during the excavation campaign in the Church of the Conversion of Saint Paul, in Roccapelago (Modena, Northern Italy; Figure 1A), a hidden crypt was discovered. The archaeological excavation was conducted between 2009 and 2011, and led to the recovery of the remains of more than 400 individuals from the inside of the crypt, most of which incomplete or commingled.

This crypt was used as a cemetery by the inhabitants of the village of Roccapelago between the 16th and the 18th centuries. The significance of this discovery lies in the large number of individuals retrieved that constitute a rare example of the natural conservation of an entire mountain community spanning 2 centuries. Thanks to such peculiarities, these important remains have been the subject of numerous anthropological and paleopathological studies (Traversari et al., 2016, 2019; Figus et al., 2017; Lugli et al., 2017, 2018; Paudice et al., 2021).

In this study, the mummified remains of Roccapelago were collected in order to examine population dynamics and relatedness between centuries in this small mountain village. For its peculiar isolated environment, Roccapelago population seems to possess the features of a genetic isolate, useful for population genetics, biomedical research, and genealogical analyses. The selection of both ancient and present-day samples was guided by anthropological, archaeological, and historical analyses. Contextually to the anthropological study, essential to acquire biodemographic and anthropometric data, it was also possible to analyze the parish registers of the church. The analysis of these documents was of fundamental importance to identify the surname lines present since the 16th century and currently still represented on the territory. Forensic and archaeogenetic protocols were thus applied to the selected individuals for the genetic investigation taking advantage of the increased analytical systems.

Research on ancient and forensic DNA are related since they deal with similar challenges, although, they are guided from different purposes and usually they rely on different methodological approaches (Hofreiter et al., 2020). Despite the progress made in recent years in the development of technologies and protocols, the selection of a suitable source of endogenous DNA is still crucial for the success of genetic analyses on degraded samples (Gonzalez et al., 2020). In particular, in ancient samples it has been demonstrated that the inner ear cochlear region of the otic capsule of the petrous bone, can lead to endogenous DNA yields up to 50–100-fold higher than those obtained from other bones, such as teeth or long bones (Gamba et al., 2014; Pinhasi et al., 2015).

Y-chromosome short tandem repeats (Y-STRs) are widely used for forensic and anthropological applications, like paternal kinship analysis, familial searching, and genetic identification of human remains (Coble et al., 2009; Haas et al., 2013; Kyser, 2017), but also for discerning the origins of migration routes and demographic processes that occurred in historical and prehistoric times (Jobling and Tyler-Smith, 2003; Larmuseau and Ottoni, 2018). The Yfiler Plus multiplex kit consists of 27 STRs including highly polymorphic loci and seven well characterized rapidly mutating markers (RM-YSTRs) with mutation rates upward of 1% (Gopinath et al., 2016), that can provide an increased power of discrimination and improve the level of paternal lineage differentiation (Olofsson et al., 2015; Rapone et al., 2016). Moreover, due to the new primer design and master mix optimization, the Yfiler Plus is designed for efficient amplification of extracted DNA casework samples, like degraded and low-template DNA.

Combining a multidisciplinary approach and following the criteria of ancient DNA (aDNA) analysis, results of Y-STRs markers are presented here with the aim to undertake a diachronic analysis of population genetics of Roccapelago community from 16th century until today. The aims of this study were to predict Y-chromosome paternal haplogroups and to assess genetic continuity between ancient mummies and the current population of Roccapelago. Moreover, this study aimed at testing and combine archaeogenetic and forensic protocols, criteria and approaches, in order to maximize the yield of endogenous DNA, obtain more likely complete Y-STR profiles and minimize exogenous contamination.

## Materials and Methods

### The Mummies and the Anthropological Analyses

The large amount of human remains retrieved in the crypt, mostly consisted of commingled and completely skeletonized human remains, comprised some mummified corpses, especially among the most recent stratigraphic units (SU; Figure 1B). Several remains were partially mummified, as a result of a natural process due to the particular microclimatic conditions of the environment. The bodies, piled in a pyramid shape, were divided into three SU: SU 28 has returned very few individuals and was dated between the end of the 16th century and the beginning of the 17th century; SU 26 was placed at the second half of the 17th and the beginning of the 18th century; SU 23, the most recent, dated at the 18th century (Figure 1C). The chronological division was made through the study of the clothing and grave goods. Sex estimation was performed by observing morphological characteristics of the skull (Acsádi and Nemeskéri, 1970). Only individuals classified as males were collected for the purposes of this study. For Y-STR analysis, we chose 25 individuals, entirely or partially skeletonized, which, among the other skeletal elements, presented the skull intact or partially preserved, but containing the portion of pars petrosa. They included two samples from SU 28, 11 from SU 26, and 12 from SU 23 (Table 1). For assessment of age at death, we combined different methods, obtaining the smallest range possible: the dental attrition was evaluated by approach of Lovejoy (1985); methods of Acsádi and Nemeskéri (1970) and Meindl and Lovejoy (1985) were used for judging cranial sutures degree of closure.

**Table 1:** List of ancient and modern samples analyzed in this study.

Ancient mummies	SU	Age at death	Powder sampled (mg)		Modern individuals	Surname code
23-41	SU23	40–49	73		3B	S1
23-1	SU23	30–39	48		4A	S2
23-12	SU23	40–49	57		10A	S2
23-31	SU23	28–29	40		16	S3
23-42	SU23	40–49	83		5A	S4
23-46	SU23	30–39	53		12A	S5
23-06	SU23	30–39	77		9A	S5
23-7	SU23	40–49	48		17A	S6
23-44	SU23	40–49	91		11A	S7
23-27	SU23	>50	76		2A	S8
23-30	SU23	30–39	40		15A	S9
23-38	SU23	40–49	80		18	S9
26-05	SU26	30–39	46		7A	S9
26-32	SU26	40–49	56		21A	S9
26-43	SU26	>50	70			
26-42	SU26	40–49	70			
26-49	SU26	30–39	73			
26-1	SU26	>50	46			
26-10	SU26	40–49	80			
26-50	SU26	>50	50			
26-12	SU26	30–39	86			
26-24	SU26	30–39	55			
26-53	SU26	>50	42			
28-02	SU28	40–49	74			
28-05	SU28	30–39	85			

*For each category the SU and the code of the founder surname (S) are reported, respectively*.

### The Selection of Modern Samples

The 14 modern samples, representing about half of the present-day population of Roccapelago, were selected by considering both the founder surnames of the village, identified thanks to the study of parish registers (that became mandatory in Italy for baptisms and marriages in 1563 after the Council of Trent and in 1614 for deaths when their rules of compilation were as well regulated by the Church), and the grandparent’s criterion.

Every single death certificate was recorded on a table in chronological order. Subsequently, the individual surnames were isolated and analyzed in terms of continuity and frequency; finally, it was possible to extract only the surnames that have demonstrated a continuous presence in the area. Collaterally, the analysis highlighted some cognominal lines that have met with real extinctions on the territory, a phenomenon perhaps due to migrations of entire families.

Modern individuals were sampled by means of cotton buccal swabs, after the collection of informed consent. The study was approved by the Bioethical Committee of the University of Bologna (IRB approval date: April 8th, 2013) and was conducted in agreement with the principles of the WMA Declaration of Helsinki.

### DNA Extraction

DNA extraction of ancient specimens was carried out in clean-rooms reserved for the analysis of degraded DNA at the Laboratory of ancient DNA (aDNALab) of the Department of Cultural Heritage, University of Bologna, Ravenna Campus. This pre-PCR area of the laboratory is physically isolated and is equipped with positive air pressure with HEPA filters and laminar flow cabinets, reserved for the different phases of the work. All steps were conducted under strict guidelines for the monitoring of the exogenous contamination and reproducibility of data (Cooper and Poinar, 2000; Gilbert et al., 2005; Llamas et al., 2017). Blanks as negative controls were processed in parallel with the samples, in order to monitor for the presence of contaminations.

In order to maximize the results of the study, the sampling was directed at the inner ear cochlear region of the petrous bone that provides good percentages of endogenous DNA (Gamba et al., 2014; Pinhasi et al., 2015). The sampling was conducted on 24 complete skulls and one isolated petrous bone (sample 26-5). For complete skulls, sampling was performed using the Cranial Base Drilling Method (CBDM; Sirak et al., 2017) in order to minimize structural damages to the cranial features. The isolated petrous bones were directly sampled on the dense parts around the cochlea (Orfanou et al., 2021). Between 40 and 91mg of bone powder were collected from each individual (Table 1) and processed with a silica-based protocol (Dabney et al., 2013; Damgaard et al., 2015), slightly modified as in Cilli et al. (2020). Samples were predigested in 1ml of Extraction buffer (0.5M EDTA, 0.25mg/ml Proteinase K, 0.05% Tween20) for 15min at 37°C to increase the endogenous DNA content and then digested overnight in a new aliquot of 1ml of the same buffer. Supernatant was centrifuged and DNA bound into silica columns with PB buffer (Qiagen). Silica columns were finally washed twice with PE buffer (Qiagen) and eluted in 50μl of elution buffer.

The DNA extraction of modern samples was performed by using the QIAmp DNA Mini kit (Qiagen), following manufacturer instructions.

### DNA Quantification and Y-STR Genotyping

After the extraction, all samples were quantified with the Quantifiler® Trio DNA Quantification Kit (Applied Biosystems) following the manufacturer’s instructions (Vernarecci et al., 2015). Quantitative PCR (qPCR) was performed in double using QuantStudio 5 Real-Time PCR System (Applied Biosystems) and data were analyzed using the HID Real-Time PCR Analysis Software v1.3 using the default settings. The quantification data obtained from the small autosomal probe of the Quantifiler Trio Kit was used for determining the concentration of aDNA.

Y-chromosome short tandem repeats amplification was performed with the Yfiler® Plus PCR Amplification Kit (Applied Biosystems), including the 27 STRs loci DYS19, DYS385a/b, DYS389I/II, DYS390, DYS391, DYS392, DYS393, DYS437, DYS438, DYS439, DYS448, DYS456, DYS458, DYS635, YGATAH4, DYS460, DYS481, DYS533, DYF387S1a/b, DYS449, DYS518, DYS570, DYS576, and DYS627, on a Veriti® 96 Well Thermal Cycler System (Applied Biosystems) and following the manufacturer’s instructions. PCR products were electrophoresed on a SeqStudio Genetic Analyzer (Applied Biosystems) and the fragment analysis was performed by GeneMapper® ID-X Software v1.6 (Applied Biosystems). Only peaks above 50 RFU were considered and replicates were performed for all ancient samples.

### Statistical Analyses

Allele frequencies, Y-chromosome diversity indexes and between-population pairwise distances were calculated with the Arlequin software version 3.5 (Excoffier and Lischer, 2010). In particular, the gene diversity (GD) for each locus was calculated using the formula GD=*n*(1−*∑x_i_*^2^)/(*n*−1), with *x_i_* being the frequency of the *i*th allele and *n* the sample size of each considered population. The intra-population haplotype diversity (HD) was estimated as HD=*n*(1−*∑p_i_^2^*)/(*n*−1), where *n* is the number of analyzed samples and *p_i_* the frequency of the *i*th haplotype. The haplotype match probability (HMP) was calculated using the formula HMP=*∑p_i_^2^*, whereas the discrimination capacity (DC) was obtained by dividing the total number of observed haplotypes for the total number of individuals in each dataset.

The obtained Y-chromosome STR haplotypes were used to infer haplogroups of individual samples by using the Whit Athey’s Haplogroup Predictor software (Athey, 2006) and the Nevgen prediction tool,^1^ while the genetic relationships between Y-STR haplotypes were explored by means of a Median Joining (MJ) Network analysis using the Network 10.2 program.^2^

For all statistical analyses, the locus DYS389II was converted to the DYS389b nomenclature by subtracting the repeat number of the DYS389I from the detected DYS389II allele, and each of both the DYS385 and DYF387S1 multi-locus markers were treated as two separated allelic series.

In order to investigate the possible genetic relationships between ancient and modern individuals and assess genetic continuity in the population of Roccapelago over about 4 centuries, we estimated the Time to the Most Recent Common Ancestor (TMRCA) for each ancient-modern pair by adopting the Walsh (2001) method as implemented in the R script from Boattini et al. (2019). For each tested pair, the input parameters required by the script are (i) the mutation model (either the stepwise mutation model – SMM or the infinite allele model – IAM, originally implemented by Walsh, 2001); (ii) the average mutation rate for the set of considered Y-STRs markers; (iii) the lambda prior hyper-parameter (Walsh, 2001); and (iv) a vector of per couple differences for the analyzed loci (i.e., a file indicating matching “0” and non-matching “1” Y-STR alleles for the IAM model, or the number of repeat differences for each locus in the case of SMM model). Data output for each tested pair returns the posterior distribution of the estimated TMRCA with mean, median, and mode values as well as the computed confidence interval(s). Comparisons between documented genealogical pairs and the TMRCA estimates obtained with the Walsh method in Boattini et al. (2019), showed that the IAM model yielded the best performance compared to SMM. In addition, among the considered summary statistics, the modal value of the posterior distributions was found to be the one best approximating the observed values. Accordingly, the IAM model was therefore chosen also in the present analysis, while the Y-STR mutation rates adopted in the procedure were taken from Ballantyne et al. (2010). Since the Walsh method implemented in Boattini et al. (2019) was originally designed for estimating TMRCA of genealogical pairs of individuals whose life span overlaps, in the present study, where pairs are instead composed by chronologically distant pairs of ancient and modern individuals, we adapted TMRCA to calculate the total number of generations separating them, which corresponds to the double of the originally provided estimate. Finally, the obtained numbers of generations were converted in years before present by using an average generation time of 33years, in agreement with genealogical-based estimates obtained from previous studies on the same geographic area (Boattini et al., 2014, 2019; Sarno et al., 2021).

## Results

### Authenticity of the Results and Success Rate of Ancient DNA Analysis

The strict criteria followed in this study allowed us to exclude any modern DNA contamination and confirm the reliability of the genotyping results. No contamination was observed in any of the blank extractions or negative PCR controls included in each reaction. Moreover, the data were consistent between replicates of amplifications. In addition, the haplotypes of the samples were different from those of the anthropologists and geneticists involved in the sampling and DNA analyses.

DNA extraction from petrous bone yielded endogenous DNA by qPCR analysis ranging from 0.004 to 0.226ng/μl for the small autosomal target and from 0.001 to 0.175ng/μl for the Y-target. The range of the degradation index (DI) spanned between 2 and 107, except one sample for which the DI could not be determined as the large autosomal target was not detected (Table 2). Furthermore, for two samples (26-12 and 26-53) the Y-target was not determined, and therefore they were not included in the amplification session.

**Table 2:** Quantification by real-time PCR and Y-chromosome short tandem repeat (Y-STRs) amplification results from the 25 mummies analyzed.

Ancient mummies	Quantity mean (ng/μl)			DI	No. STR Yfiler plus/27
	RT_Trio L-target	RT_Trio S-target	RT_Trio Y_target		
23-41	0.0052	0.1127	0.0875	21	25
23-1	0.0003	0.0221	0.0146	82	16
23-12	0.0051	0.0543	0.0377	11	24
23-31	0.0074	0.0226	0.0153	3	27
23-42	0.0020	0.1186	0.0905	61	21
23-46	0.0313	0.2144	0.1714	7	27
23-06	0.0064	0.0473	0.0470	7	25
23-7	0.0107	0.0344	0.0236	3	27
23-44	0.0017	0.0866	0.0819	50	24
23-27	0.0038	0.0366	0.0006	9	neg
23-30	0.0037	0.0354	0.0005	9	neg
23-38	0.0003	0.0159	0.0112	107	7
26-05	0.0214	0.2256	0.1753	10	27
26-32	0.0133	0.0288	0.0169	2	27
26-43	0.0021	0.0776	0.0498	37	24
26-42	0.0064	0.0261	0.0241	4	24
26-49	0.0012	0.0202	0.0143	21	14
26-1	0.0051	0.0761	0.0748	15	25
26-10	0.0041	0.0289	0.0217	7	25
26-50	0.0021	0.0115	0.0061	7	21
26-12	0.0013	0.0311	n.d.	25	neg
26-24	0.0003	0.0037	0.0027	13	neg
26-53	0.0023	0.0227	n.d.	9	neg
28-02	n.d.	0.0151	0.0095	n.d.	10
28-05	0.0002	0.0168	0.0147	104	16

*The total amount of quantified DNA for large and small autosomal, and Y targets, the degradation index (DI) and the total of alleles detected for each petrous bone are reported*.

Yfiler Plus STRs markers were successfully amplified for at least 10 loci in 19 out of 25 ancient DNA samples and, as expected, in all the 14 analyzed modern individuals from Roccapelago (Table 2). In particular, only five of the analyzed ancient specimens (namely samples 23-7, 23-31, 23-46, 26-05, and 26-32) provided complete results for all the 27 loci of the Yfiler Plus; for the remaining genotyped mummies partial Y-chromosome haplotypes were instead obtained consisting of 25 (samples 23-06, 23-41, 26-1, and 26-10), 24 (samples 23-12, 23-44, 26-42, and 26-43), 21 (samples 23-42, 26-50), 16 (samples 23-01, 28-05), 14 (sample 26-49), and 10 (sample 28-02) STR markers, respectively. Not surprisingly, the two human remains from the oldest stratigraphic unit (i.e., SU28) showed the lowest number of genotyped Y-STRs loci. The remaining amplified ancient samples provided less than nine Y-STRs loci and therefore were not considered for the analyses.

All samples provided results for loci DYS385, DYS393, DYS456, DYS458, DYS460, DYS570, and DYS576, while the worst performance was obtained for the largest loci DYS391 and the DYS533. We also observed a duplication (alleles 14 and 15) at the locus DYS19 in sample 26-10 confirmed by PCR replicates. In Supplementary Table S1, all observed Y-STRs haplotypes in ancient and modern individual sample are provided. When comparing haplotypes of present-day individuals sharing the same surnames, one step mutations in two groups of modern samples for three of seven RM Y-STRs included in the kit were found, in particular for DYS627 in two individuals and for DYS570 and DYS518 in four modern samples.

### Haplotype Diversity Analysis and Forensic Indexes

Being aware that the presence of some missing loci in ancient individuals may affect the power of discrimination in distinguishing haplotypes, in order to maximize as much as possible the level of male lineage differentiation we considered for statistical analyses only individuals with no more than three missing genotyped loci, thus resulting in 13 ancient and all 14 modern samples left for subsequent computations. On the whole, using the Y-STR Yfiler Plus Kit a total of 20 unique haplotypes were observed among the 27 considered individuals of Roccapelago (Table 3), with two haplotypes instead occurring twice and another haplotype shared among three samples. In particular, two modern individuals (4A and 10A, who also share the same surname), were found to share a complete Yfiler Plus profile. The ancient mummy 23-44 instead matches for its 24 genotyped loci the ones of the modern individual 7A. Finally, also the ancient sample 23-7 and the modern individual 17A shared an identical complete Yfiler Plus haplotype, which also matches the one of the mummy 23-06 for its 25 genotyped loci.

**Table 3:** Diversity indexes for the whole analyzed samples from Roccapelago, and for the ancient (RP_A) and modern (RP_M) population sets, separately.

Observed haplotype	Total	27	RP_A	13	RP_M	14
	Yfiler	Yfiler Plus	Yfiler	Yfiler Plus	Yfiler	Yfiler Plus
Once	9	20	5	11	7	12
Twice	1	2	1	1	2	1
Three times	1	1	2	0	1	0
Four times	2	0	0	0	0	0
Five times	1	0	0	0	0	0
HD	0.9259	0.9858	0.9103	0.9872	0.9451	0.9890
HMP	0.1084	0.0508	0.1598	0.0888	0.1224	0.0816
DC	0.5185	0.8519	0.6154	0.9231	0.7143	0.9286

*HD, haplotype diversity; HMP, haplotype match probability; and DC, discrimination capacity*.

Haplotype diversity and DC for the Yfiler Plus Kit were found to be 0.9858 and 0.8519, respectively in the ancient-plus-modern dataset. Comparable, if not higher values were observed also when the 13 ancient (RP_A: HD=0.9872 and DC=0.9231) and the 14 modern (RP_M: HD=0.9890 and DC=0.9286) individuals were considered as two separated population sets. In all of the cases, the use of the Yfiler Plus Kit was proven to significantly increase the power of discrimination compared to the original Yfiler panel (Table 3).

In particular, the 10 Y-STRs loci added to the 17-plex Yfiler kit, and especially the seven newly-included RM loci, were indeed the ones showing on average the higher values of gene diversity (GD>0.70) in all the considered population sets (Table 4)

**Table 4:** Genetic diversity for each Y-STR marker in the considered datasets (RP_T: whole samples from Roccapelago; RP_A: ancient mummies; and RP_M: modern individuals).

	Gene diversity (GD)				
	RP-T	RP-M	RP-A		
DYS19	0.52	0.527473	0.530303	**Y FILER 17**	**Y FILER PLUS**
DYS385a	0.698006	0.703297	0.692308		
DYS385b	0.777778	0.802198	0.75641		
DYS389I	0.54416	0.604396	0.512821		
DYS389b	0.64	0.648352	0.581818		
DYS390	0.717949	0.692308	0.730769		
DYS391	0.5671	0.604396	0.464286		
DYS392	0.453333	0.527473	0.327273		
DYS393	0.581197	0.582418	0.5		
DYS437	0.655271	0.648352	0.705128		
DYS438	0.643875	0.659341	0.653846		
DYS439	0.612536	0.615385	0.5		
DYS448	0.5	0.494506	0.545455		
DYS456	0.632479	0.648352	0.653846		
DYS458	0.735043	0.736264	0.74359		
DYS635	0.772308	0.835165	0.712121		
YGATAH4	0.492308	0.527473	0.484849		
**Average Y Filer 17**	**0.620196**	**0.638655**	**0.593813**		
DYS460	0.501425	0.538462	0.461539		
DYS533	0.571429	0.615385	0.476191		
DYS481	0.737892	0.769231	0.730769		
DYS576	0.735043	0.692308	0.730769	**RM LOCI**	
DYF387S1a	0.621083	0.604396	0.653846		
DYF387S1b	0.723647	0.747253	0.717949		
DYS449	0.757835	0.747253	0.794872		
DYS518	0.76	0.758242	0.818182		
DYS570	0.700855	0.78022	0.653846		
DYS627	0.621083	0.659341	0.602564		
**Average additional Y plus loci**	**0.673029**	**0.691209**	**0.664053**		
**Average RM loci**	**0.702792**	**0.712716**	**0.71029**		

*All samples with more than three missing genotyped loci were excluded from the computations. Both the DYS385 and DYF387S1 multi-locus markers were considered as separated loci*.

### Population Relationship Analyses

Y-chromosome short tandem repeat haplotypes were used to predict Y-chromosome paternal haplogroups and to assess genetic continuity between ancient mummies and the current population of Roccapelago.

Y-chromosome haplogroups were successfully inferred for all samples, with high prediction probabilities in almost all of the cases. Lower probabilities were observed for those samples showing more than 10 missing loci (Supplementary Table S1). Ancient DNA samples were found to belong to the Y-chromosome macro lineages I2a, J1, J2b, and R1b. The same haplogroups also characterize the present-day individuals, suggesting a homogenous Y-chromosome composition with respect to the current population of Roccapelago, even when using a Y haplogroup predictor with a higher level of resolution. Accordingly, the degree of differentiation between RP_A and RP_M population sets, evaluated based on both Y-chromosome haplogroup frequencies and Y-STR RSTs genetic distances, resulted low and not significant (FST: 0.00381, *p*: 0.41877 and RST: 0.01730, *p*: 0.28433, respectively).

**Figure 2:**
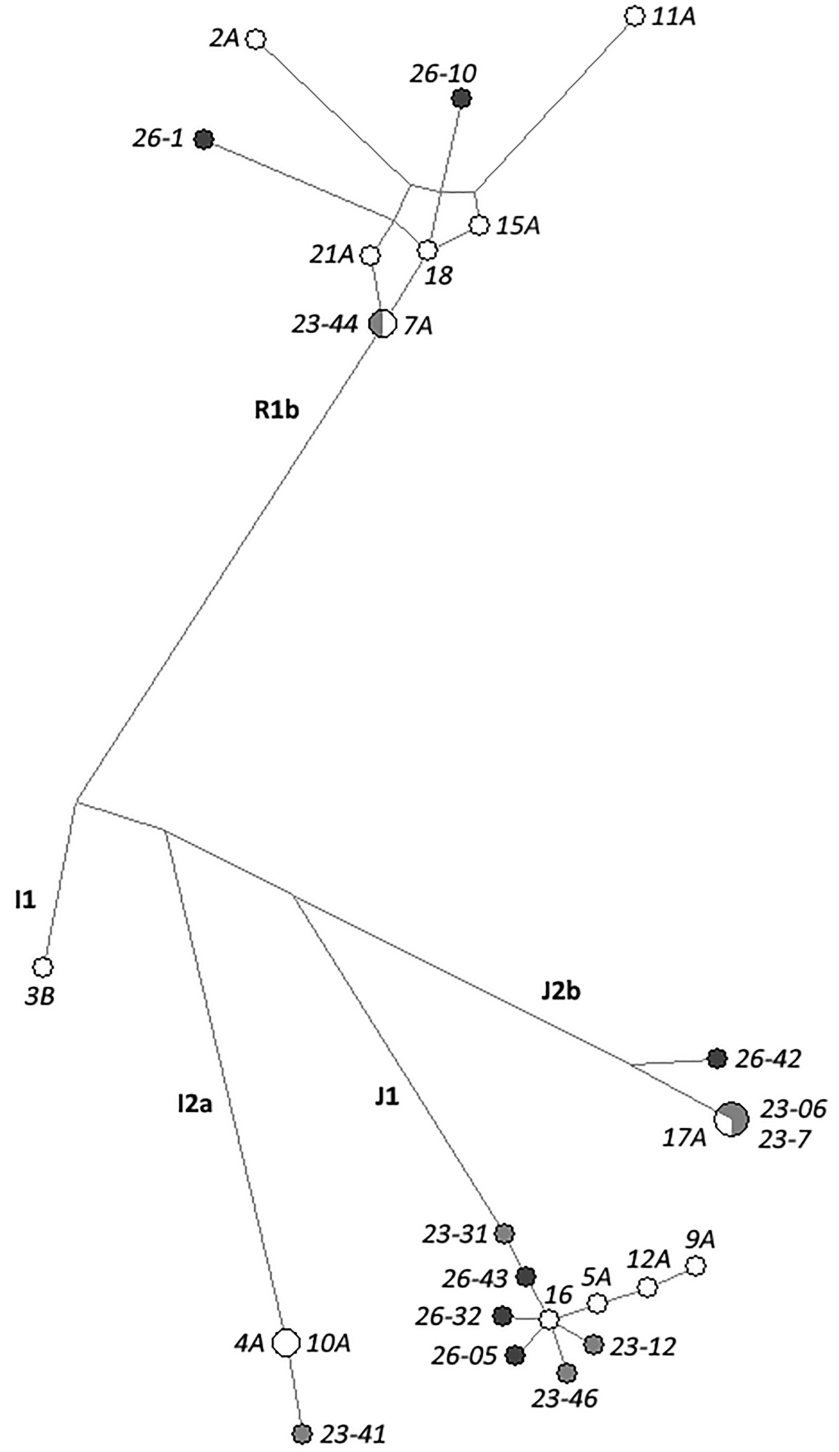
Median Joining (MJ) network analysis of the Y-chromosome haplotypes based on the 27 Yfiler Plus STRs in the 13 ancient and 14 modern samples from Roccapelago. Present-day individuals are highlighted in white dots, ancient mummies from the stratigraphic units SU23 in light-gray and those from the SU26 in dark-gray.

The phylogenetic relationships between modern and ancient haplotypes (including only individuals showing no more than three missing loci as above) were visually represented by means of a MJ Network (Figure 2). As expected, haplotypes clustered based on corresponding haplogroup lineage, also showing little differences in STR profiles between the ancient and modern individuals of Roccapelago within the same haplogroups. In particular, the haplotype shared between the modern 4A and 10A samples, also shows a single different locus with respect to the 25 markers successfully typed in the ancient mummy 23-41. Analogously, all the six J1 ancient samples (23-31, 23-12, 23-46, 26-05, 26-32, 26-43) appear one-step or two-steps neighbors of the modern J1 sample 16. The ancient J2b mummies 23-7 and 23-06 (successfully typed for all the 27 and for 25 loci, respectively) share an identical Y-chromosome haplotype with the modern individual 17A, as outlined also above by the haplotype sharing analysis. Similarly, the 24 genotyped loci of the ancient mummy 23-44 match the haplotype of the modern individual 7A, further showing one or at most two different loci also with respect to the other modern R1b individuals who share the same surname of 7A (namely 15A, 18, and 21A).

**Figure 3:**
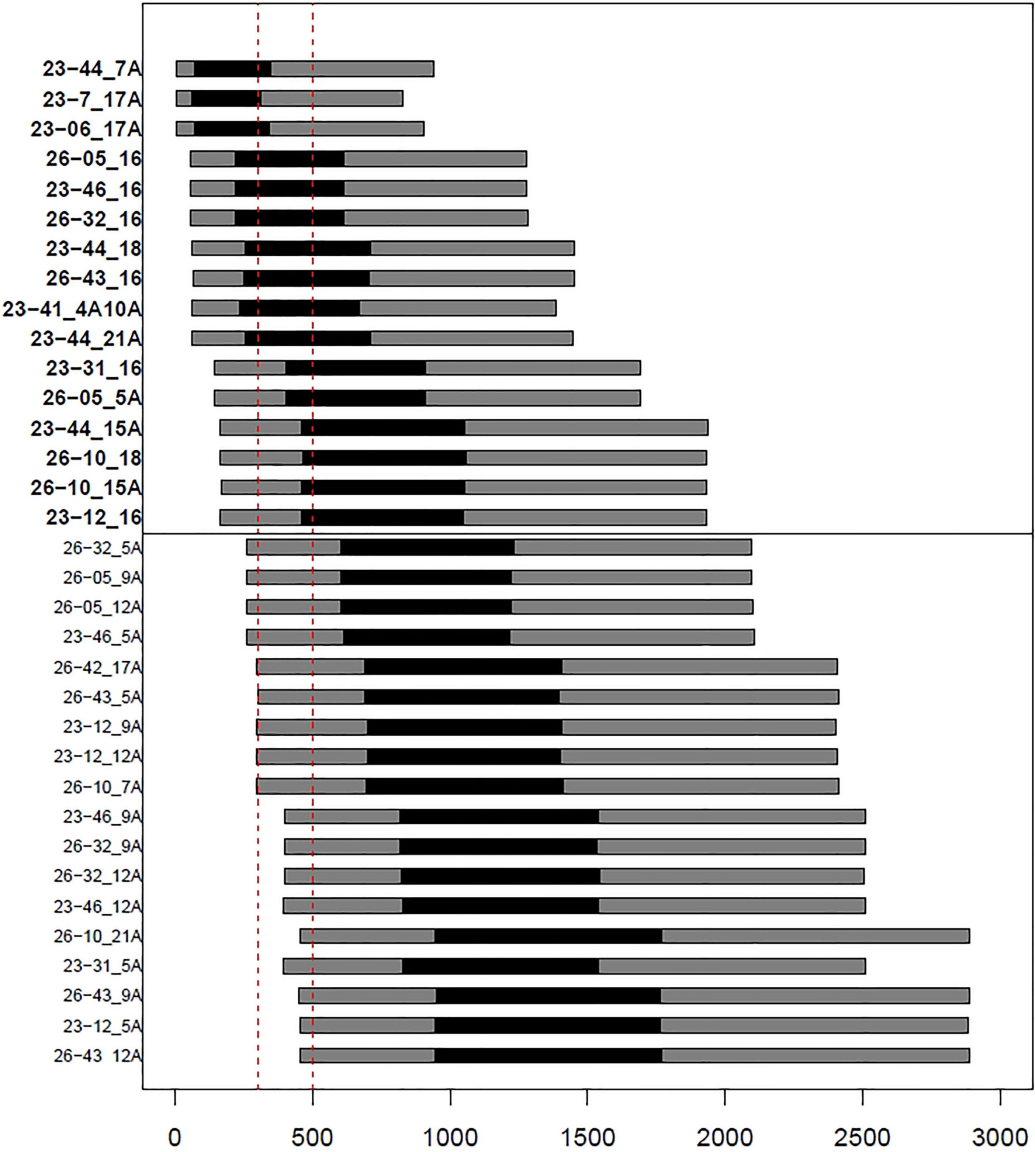
Barplots representing confidence intervals (black: 50% CI; gray: 95% CI) of estimates of times separating ancient and modern samples. Vertical dashed lines (in red) delineate the chronological window of Roccapelago mummies (~300–500years ago). The upper panel shows pairs with best-matching results (i.e., overlap between mummies chronological window and the 50% CIs of time estimates).

In order to formally assess the presence of genetic connections among the ancient mummies and the present-day inhabitants of Roccapelago, we estimated the expected TMRCA between ancient-modern pairs (Supplementary Table S2) by exploiting the Bayesian procedure originally designed by Walsh (2001) and implemented by Boattini et al. (2019), as detailed in Materials and Methods. In particular, we looked for pairs for which the CI of the inferred values fall within the chronological range of dating of the ancient mummies, by specifically retaining those (highlighted in bold in the upper panel of Figure 3) whose more stringent 50% CI overlaps with the time window of 300–500years ago. Accordingly, we observed that 11 of 13 mummies indeed showed signs of continuity with modern inhabitants of Roccapelago (Figure 3), thus suggesting that the genetic composition of the population did not significantly change with time. Interestingly, significant TMRCA results emerged for the same couples also highlighted in the Network analysis (i.e., 23-41 with 4A/10A; 23-12, 23-31, 23-46, 26-05, 26-32, and 26-43 with 16A; 23-06 and 23-7 with 17A; and 23-44 with 7A, 21A, 18, and 15A).

## Discussion

In forensic and archaeological applications, DNA degradation represents a challenge during each phase of the analyses, and methodological improvements are necessary to obtain successful results from heavily degraded and low template DNA samples like mummies. Certainly, in the last years, the scientific progress and technological development contributed to overcome the difficulties to obtain more likely complete STR profiles for genetic identification, and at the same time the standard molecular biology approaches have been widely improved to address issues of DNA degradation, contamination, and inhibition (Malmström et al., 2005; Votrubova-Dubska et al., 2016).

Among the methodological innovations that have made possible to overcome these difficulties there is the identification of the best skeletal elements, of which in primis petrous bones (Edson et al., 2009; Gamba et al., 2014; Pinhasi et al., 2015). Even though, this skeletal element can lead to endogenous DNA yields that are up to 50–100-fold higher than those obtained from other bones, recent studies demonstrated that other samples (*in situ* molars, thoracic vertebrae, talus, and distal phalanx) can yield adequate DNA for most applications in human palaeogenetics (Higgins et al., 2013; Hansen et al., 2017; Parker et al., 2020), especially ear ossicles (Sirak et al., 2020). Important methodological improvements also regarded the development of increasingly efficient extraction protocols and genotyping kits. The petrous bone (the densest part of the temporal bone) is a source of DNA in both larger quantities and of better quality than other bones, and it represents the substrate of choice for DNA extraction and analyses of ancient and/or degraded DNA (Gonzalez et al., 2020). Although, it was originally published in the forensic field (Edson et al., 2009), the petrous bone was not truly implemented in laboratory work by the forensic community, probably due to ethical aspects related to the invasive sampling (Hofreiter et al., 2020). Recently, several studies have however demonstrated the usefulness of this skeletal element to genotype forensic samples (Pilli et al., 2018; Gonzalez et al., 2020), and the improving Y-STR typing for human identification by the YFiler Plus kit in skeletal remains (Ambers et al., 2018). In addition, the increased efficiency of extraction protocols and amplification systems should also be noted (Loreille et al., 2007; Amory et al., 2012; Dabney et al., 2013; Hofreiter et al., 2020).

This study confirmed the value of the otic capsule of the petrous bone as a skeletal element in maximizing DNA yield in order to increase the probability of obtaining complete Y-STR profiles and limit allelic drop-out, but also in preventing contamination with exogenous human DNA (Gonzalez et al., 2020). The genetic material showed different rates of preservation, ranging from a moderate to a significant degradation (reaching 107), as indicated from the values of the DI reported in Table 2. In only one sample the large autosomal target was not detected. Although, it is well-known that physical, biological, and chemical factors are deeply involved in the long-term preservation of DNA (Collins et al., 2002), in our results the typing success reached 76% and PCR negative samples were related to the small detected quantity of Y DNA. For the two samples completely negative for the Y-target quantification, the results could also be attributed to a not certain morphological sex attribution since a minimal quantity of autosomal DNA was detected. Moreover, in ancient samples showing similar DNA quantity, a strong correlation between DI values and typing success was not observed.

Specifically, 19 out of 25 ancient DNA samples showed more than 10 loci typed. A total of five specimens provided a complete profile for all the 27 loci of the Yfiler Plus kit, while for the other samples it was possible to obtain a number of amplified loci from 25 to 10, where the worst results were obtained from the oldest stratigraphic unit (i.e., SU28). Regarding the STR markers, our typing data suggest that for the analysis of skeletal remains a better performance can be obtained for the DYS385a/b, DYS393, DYS456, DYS458, DYS460, DYS570, and DYS576 loci but not for DYS391 and DYS533 loci, possibly due to the new primer design resulting in a larger amplicon size in the YFiler Plus kit compared to Yfiler kit. Indeed, although, a multiplex system with a greater number of markers increases discriminatory power, often aDNA is limited in quantity and compromised quality resulting in reduction or loss of signal especially for larger loci.

Among the modern individuals sharing the same surnames a one-step mutation was observed in at least one RM Y-STR (DYS570, DYS627, and DYS518). This finding supports again the potentially increased mutability of these markers, which would ideally become the first choice for Y-STR testing in forensic casework (excluding family testing) due to their superior value in paternal lineage differentiation as well as male relative separation (Ballantyne et al., 2012; Boattini et al., 2016). The data also confirmed that the YFiler Plus kit offers higher power of discrimination and enhanced chemistry for improved performance with challenging samples, as well as a higher resolution than the YFiler panel, especially thanks to the seven newly-included RM loci that on average showed the higher values of gene diversity (Table 4). This agrees with previously published data (Olofsson et al., 2015; Rapone et al., 2016; Fan et al., 2019). The Yfiler Plus ability to discriminate among closely related males could have useful applications especially in situations of multiple victims from the same family buried together, like a mass grave, or mummies discovered in a crypt (Ambers et al., 2018), by however considering the possibility of false exclusions due to mutation when compared to living relatives in kinship testing.

As concern population relationships analyses, overall our results support a paternal genetic continuity of the analyzed population from the 16th century until today. In general, the predicted Y-chromosome haplogroups reflect lineages commonly observed in Italy (Grugni et al., 2018) and revealed a homogeneous genetic composition through centuries between the ancient and modern samples of Roccapelago, as supported even by both Y-chromosome haplogroup frequencies and RSTs genetic distances. The connection between ancient and modern individuals of Roccapelago is well documented also in the network analysis (Figure 2), where both mummies’ and present-day haplotypes clustered together based on haplogroups assignment. Additionally, searching against the Y-haplotype reference database (YHRD) resulted in non-matches between each ancient sample and any of the other Yfiler Plus reference samples included in the database (encompassing also other Italian groups), thus providing further evidence of a closer proximity between the mummies’ haplotypes and the modern individuals of Roccapelago. With the aim to formally test the genetic connection among the ancient mummies and the present-day inhabitants of Roccapelago, we finally estimated the expected TMRCA between them. This analysis, supported by the network results, confirmed signals of genetic continuity between ancient and modern inhabitants of Roccapelago for 11 out of the 13 mummies here considered.

These findings agree with the historical data and the partial past geographical isolation of this small village. In fact, from the analysis of parish registers it has been highlighted, during centuries, a trend toward exogamic marriages, with external male contributions, mainly from the areas interested by transhumance. However, for the founding surnames, like the ones sampled for this study, a tendency to isonymia and consanguinity has been noted from the parish registers. This probably was due to a social-cultural practice to pass down lands through generations between the same ancient founder families (Traversari, 2020).

In conclusion, a multidisciplinary approach was successfully applied to the mummies of a small mountain community dated to 16th–18th centuries, whose Y-STR haplotypes were analyzed and compared to data obtained from the modern people of the village. We combined forensic genetics and archaeogenetics approaches and protocols with historical, archaeological, and anthropological data, in order to understand population dynamics and relatedness on this peculiar community from the 16th century. This study highlights the need of the contribution from different disciplines to a better and more efficient human identification.

## Data Availability Statement

The original contributions presented in the study are included in the article/Supplementary Material, further inquiries can be directed to the corresponding author.

## Ethics Statement

The studies involving human participants were reviewed and approved by Bioethical Committee of the University of Bologna (approval date: April 8th, 2013). The patients/participants provided their written informed consent to participate in this study.

## Author Contributions

DL and SP conceived the study. EC, MT, and CB designed the experiments. CB, FF, and EC performed the genetic analyses. SS, AB, CB, and EC analyzed the data. MT performed the archaeological, anthropological, and historical analyses. EC, SS, and CB wrote the original draft. All authors contributed to the article and approved the submitted version.

## Funding

This work was supported by the MIUR-PRIN 20177PJ9XF grant to DL.

## Conflict of Interest

The authors declare that the research was conducted in the absence of any commercial or financial relationships that could be construed as a potential conflict of interest.

## Publisher’s Note

All claims expressed in this article are solely those of the authors and do not necessarily represent those of their affiliated organizations, or those of the publisher, the editors and the reviewers. Any product that may be evaluated in this article, or claim that may be made by its manufacturer, is not guaranteed or endorsed by the publisher.

## Footnotes

^1^ https://www.nevgen.org

^2^ http://www.fluxus-engineering.com
